# Within-host microevolution of *Pseudomonas aeruginosa* in Italian cystic fibrosis patients

**DOI:** 10.1186/s12866-015-0563-9

**Published:** 2015-10-19

**Authors:** Rasmus Lykke Marvig, Daniela Dolce, Lea M. Sommer, Bent Petersen, Oana Ciofu, Silvia Campana, Søren Molin, Giovanni Taccetti, Helle Krogh Johansen

**Affiliations:** Department of Immunology and Microbiology, Costerton Biofilm Center, Faculty of Health and Medical Sciences, University of Copenhagen, Copenhagen, Denmark; Center for Genomic Medicine, Rigshospitalet, Copenhagen, Denmark; Department of Paediatric Medicine, Cystic Fibrosis Centre, Anna Meyer Children’s University Hospital, Florence, Italy; Department of Systems Biology, Technical University of Denmark, Lyngby, Denmark; The Novo Nordisk Foundation Center for Biosustainability, Technical University of Denmark, Lyngby, Denmark; Center for Biological Sequence Analysis, Technical University of Denmark, Lyngby, Denmark

**Keywords:** Bacterial pathogens, Genetic adaptation, Evolution, Cystic fibrosis

## Abstract

**Background:**

Chronic infection with *Pseudomonas aeruginosa* is a major cause of morbidity and mortality in cystic fibrosis (CF) patients, and a more complete understanding of *P. aeruginosa* within-host genomic evolution, transmission, and population genomics may provide a basis for improving intervention strategies. Here, we report the first genomic analysis of *P. aeruginosa* isolates sampled from Italian CF patients.

**Results:**

By genome sequencing of 26 isolates sampled over 19 years from four patients, we elucidated the within-host evolution of clonal lineages in each individual patient. Many of the identified mutations were located in pathoadaptive genes previously associated with host adaptation, and we correlated mutations with changes in CF-relevant phenotypes such as antibiotic resistance. In addition, the genomic analysis revealed that three patients shared the same clone. Furthermore, we compared the genomes of the Italian CF isolates to a panel of genome sequenced strains of *P. aeruginosa* from other countries. Isolates from two of the Italian lineages belonged to clonal complexes of *P. aeruginosa* that have previously been identified in Danish CF patients, and our genomic comparison showed that clonal isolates from the same country may be more distantly related than clonal isolates from different countries.

**Conclusions:**

This is the first whole-genome analysis of *P. aeruginosa* isolated from Italian CF patients, and together with both phenotypic and clinical information this dataset facilitates a more detailed understanding of *P. aeruginosa* within-host genomic evolution, transmission, and population genomics. We conclude that the evolution of the Italian lineages resembles what has been found in other countries.

## Background

Advances in high-throughput DNA sequencing techniques have made it possible to follow the within-host genomic evolution of bacterial pathogens by comparing genomes of longitudinally collected bacterial isolates sampled from human hosts [1]. The genomic information may be used to understand pathogen population diversity, host adaptation, and routes and sources of transmission.

*Pseudomonas aeruginosa* infections in cystic fibrosis (CF) patients represents an infectious disease scenario in which within-host genomic evolution of clonal lineages of infecting bacteria can be followed by making genomic comparisons of clonal isolates sampled over time [2]. A number of clinical collections of freeze-stored *P. aeruginosa* isolates from CF patients have been genome sequenced to investigate pathogen microevolution. This includes investigation of within-host evolution of *P. aeruginosa* lineages isolated from CF patients from Argentina, Canada, Denmark, Germany, United Kingdom, and United States, respectively [3–10]. Nonetheless, to obtain a more complete basis for understanding *P. aeruginosa* infections in CF patients and to facilitate comparative studies to assess the generality of findings, it is necessary to investigate more clinical collections of *P. aeruginosa*. In order to serve as references, such investigations should be as comprehensive as possible, for example by including both genomic, phenotypic, and clinical information.

Here, we report the first genomic analysis of *P. aeruginosa* isolates sampled from Italian CF patients, aiming to compare whether within-host evolution of *P. aeruginosa* in Italian CF patients resembles what has been found in other countries.

In total, we sequenced 26 isolates sampled over a period of 19 years from four different patients. Genomic comparisons showed that the 26 isolates belonged to six different clone types (clonal complexes), which we named IT01-IT06. Each clone type was found to infect a single patient, except for clone type IT05 that was shared among three patients.

We conducted both an inter-clonal genomic analysis to determine the genetic relationship between different strains, and an intra-clonal genomic analysis to reveal within-host microevolution of the individual clonal lineages. We correlated the genetic changes with changes in relevant phenotypes and patient treatment. Finally, we compared the findings from this study of evolution of *P. aeruginosa* in Italian CF patients to the findings from similar studies encompassing lineages and patient cohorts from other countries. Altogether, continued genome sequencing of bacterial pathogens will improve our understanding of within-host genomic evolution, transmission, and population genomics.

## Results

### Collection of *P. aeruginosa* isolates from Italian CF patients

**Fig. 1:**
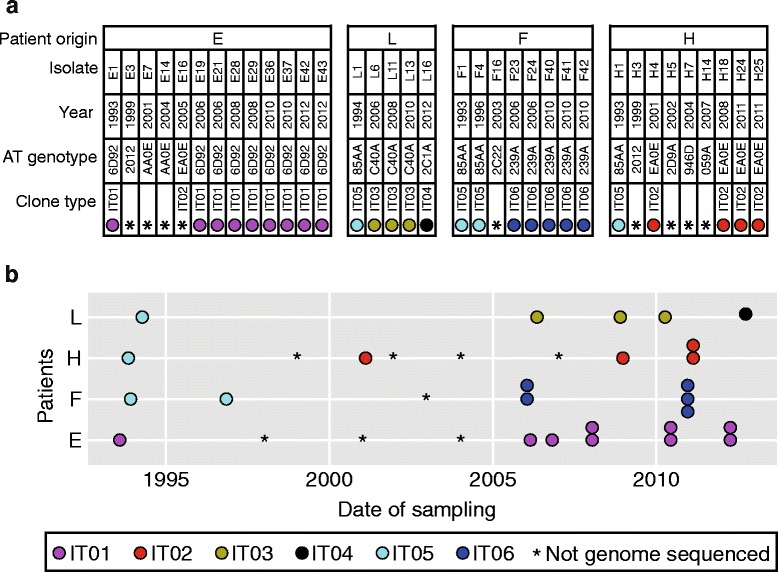
Collection of *Pseudomonas aeruginosa* isolates. **a** Information about *P. aeruginosa* isolates collected from patients E, L, F, and H. AT genotype refers to the genotype code determined by ArrayTube multimarker microarray. **b** Longitudinal overview of samples

In order to investigate the within-host genomic evolution of *P. aeruginosa* lineages infecting CF patients, we performed a retrospective study of a collection of *P. aeruginosa* isolates from four patients attending the Cystic Fibrosis Center at Anna Meyer’s Children University Hospital (Florence, Italy). The four patients named E, F, H and L were all born in the beginning of the 1990s (1991–1993), and they had their first colonization by *P. aeruginosa* within the first three years of life. The first and subsequent lung isolates of *P. aeruginosa* from the four patients were all stored to yield a collection of 35 isolates sampled over a time period of 19 years (1993 to 2012) (Fig. 1).

The ability of *P. aeruginosa* to cause infection in CF patients is not pertained to a single or a few strains (i.e. clonal complexes or clone types) of *P. aeruginosa*, and CF children are anticipated to acquire their first *P. aeruginosa* infection from environmental strains that are naïve to the human airways. Accordingly, many different and unique strains are observed to infect CF patients [9, 11–13]. While the early *P. aeruginosa* infections may possibly be eradicated, it is commonly observed that the same clone type persist in the airways of CF patients, and a single clone type is typically observed to be dominant [9]. In order to identify such persistent lineages, we typed the 35 strains using the ArrayTube multimarker microarray targeting 13 single nucleotide polymorphisms (SNPs) in the core genome and additional genetic markers in the accessory genome [13]. ArrayTube genotyping revealed that all four patients harbored reoccurring genotypes (Fig. 1). The reoccurrence of identical bacterial genotypes in the same patient likely reflect that strains persist in the airways of the patients [14]. Alternatively, the reoccurrence of identical genotypes may be due to independent re-colonization from the same environmental source.

### Genome sequencing of isolates to determine genetic relationships

To further elucidate the possibility that *P. aeruginosa* lineages persisted within the airways of the patients, we genome sequenced 26 of the isolates to investigate their genomic relationships (Fig. 1). Genome sequencing confirmed that isolates of the same genotype were closely related (i.e. isolates are of the same clonal lineage), and we name these clonal lineages IT01, IT02, IT03, IT05, and IT06. Only one isolate of ArrayTube genotype 2C1A was sequenced, and we name this strain IT04.

Isolates of the same lineage differed at most by 84 SNPs (isolates E1 and E36 sampled 17 years apart), and on average 41 SNPs separated clonal isolates. Most diversity was present among isolates of the IT01 lineage (average genetic distance between IT01 isolates: 57 SNPs), whereas almost no SNP differences were observed between isolates belonging to lineage IT05 (isolates were separated by at most 2 SNPs).

### Transmission of the IT05 lineage between patients

Lineages IT01, IT02, IT03, and IT06 were found to infect only a single patient (patients E, H, L, and F, respectively), suggesting that the patients had picked up the respective lineages from the environment. Contrary, lineage IT05 was found in both patients F, H, and L, suggesting that the IT05 lineage had spread among the patients by either direct patient-to-patient transmission or indirect transmission via a common environmental reservoir. Interestingly, the IT05 lineage is the first clone type of *P. aeruginosa* to be isolated from all three patients in years 1993, 1993, and 1994, respectively. However, in all cases other clone types of *P. aeruginosa* replace the IT05 clone type. We speculate that the IT05 lineage reside in a common environmental source to which the patients are frequently exposed, enabling the IT05 lineage to successfully colonize multiple patients, but that the IT05 lineage in the long run is replaced by other clone types that are inherently better to thrive in the patients’ airways.

### Phylogeny and mutational signatures

**Fig. 2:**
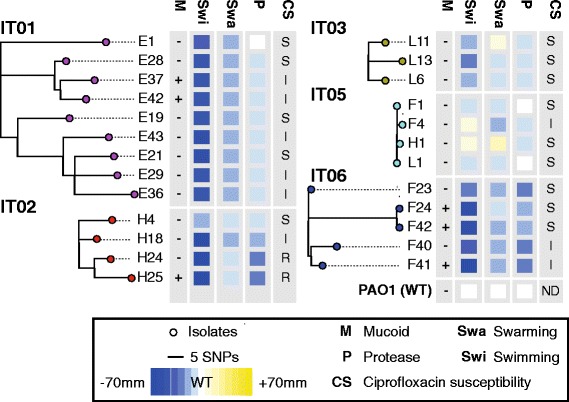
Phylogeny and selected phenotypes of *Pseudomonas aeruginosa* lineages IT01, IT02, IT03, IT05, and IT06. Maximum-parsimonious phylogenetic trees were constructed by PAUP* (Methods). Size of swimming, swarming, and protease clearing zones relative to reference strain PAO1 are indicated by colors. Presence of mucoid phenotype is denoted by ‘+’. Ciprofloxacin susceptibility is indicated by ‘S’ (>21 mm clearing zone), ‘I’ (16–20 mm clearing zone), or ´R´ (no clearing zone)

Next, we compared clonally related genomes to reconstruct the evolutionary history of each of the clonal lineages (Fig. 2). The mutations in each of the lineages accumulated in a highly parsimonious fashion (average parsimonious consistency of 0.99; Table 1), reflecting a unidirectional and clonal evolution since the most recent common ancestor. Thus, using a maximum-parsimonious phylogenetic model, we were able to make accurate inferences about the succession of mutations and the relationship among *P. aeruginosa* clones.

**Table 1:** Mutations accumulated in *Pseudomonas aeruginosa* lineages during infection

Lineage	IT01	IT02	IT03	IT05	IT06
SNPs	193	38	21	3	48
Missense	104	17	7	1	25
Nonsense	7	1	0	1	2
Silent	53	5	7	1	11
Intergenic	29	15	7	0	10
Intragenic indels	46	23	7	4	43
Intergenic indels	18	6	3	2	13
dN/dS	0.70	1.20	0.33	0.67	0.82
Probability of dN/dS = 1	3.61E-05	8.19E-01	3.95E-04	4.23E-01	1.70E-01
Maximum-parsimonious SNP events	193	39	21	3	48
Parsimony consistency	1.00	0.97	1.00	1.00	1.00

General conclusions about to what extent natural selection has been the driving force in the fixation of genetic variants, can be inferred from measuring the relative rates of nonsynonymous (dN) and synonymous (dS) genetic changes [15]. A dN/dS ratio greater than one implies that there has been an overall positive selection for mutations; whereas a ratio less than one implies that there has been a selection for removal of mutations, i.e. negative selection. However, note that the dN/dS ratio should be interpreted as an average signal of selection not giving any information on if both positive and negative selection has acted at different sites and/or times during the evolution. We found a significant signature of negative selection in lineages IT01 (dN/dS = 0.7) and IT03 (dN/dS = 0.3), whereas the dN/dS ratios in the other three lineages were neither significantly positive nor negative (Table 1). Our observed range of dN/dS from 0.3 to 1.2 is in accordance with previous findings of within-host microevolution to be affected by both negative [5, 8] and positive selection [10].

Furthermore, we performed Bayesian analyses of mutation rates, and we estimated the yearly rate of SNP mutations in IT01 lineage to be 2.7 SNPs/year (95 % highest posterior density (HPD; see Methods) 1.0–4.4 SNPs/year), which is equivalent to 4.5 × 10^−7^ SNPs/year per site. This means that the mutation rate of the IT01 lineage is almost identical to the within-host mutation rate (2.6 SNPs/year) estimated for the DK02 lineage evolving in chronically infected Danish CF patients [8]. Also, the rate is within the range of mutation rates estimated in a number of other studies: Snyder et al. reported a mutation rate of 2.4-3.0 SNPs/year during a hospital outbreak [16]; Markussen et al. found the DK01 lineage to accumulate 1.3 SNPs/year in a chronically infected Danish CF patient; and Cramer et al. estimated the mutation rate of lineage PA14 to be ~1 SNP/year over the course of infection of a German CF patient [3]. Mutation rates of the other lineages (IT02, IT03, IT05, IT06) could not be estimated since the number and temporal distribution of isolates were insufficient to obtain proper estimates of the mutation rates (effective sample sizes (ESS) of modeled parameters were below 10).

### Positive selection for mutations in pathoadaptive genes

Genetic adaptation is hypothesized to play a major role in the successful establishment of chronic *P. aeruginosa* infections of CF patients [3, 8–10, 17–22], and Marvig et al. recently performed a genome-wide mutational analysis of 36 different *P. aeruginosa* lineages to identify 52 candidate ‘pathoadaptive genes’ targeted by mutations to optimize pathogen fitness [9]. We found that 34 of 365 identified intragenic mutations were located within the 52 candidate pathoadaptive genes, corresponding to an 11-fold enrichment of mutations in these particular 52 genes relative to all other genes (*P(X ≥ 34) ~ pois(λ = 3.34)* = 7.8 × 10^−22^, where *λ* is the expected number of mutations in 52 genes). This suggests that the mutations in the pathoadaptive genes have been positively selected in the host airways. In agreement with this, we found a significant mutational signature of positive selection of SNPs in pathoadaptive genes (*P* = 0.007; dN/dS > 5; 15 nonsynonymous SNPs and none syonymous SNPs) whereas SNPs in the remainder of genes showed a signature of negative selection (dN/dS = 0.7). Mutations in pathoadaptive genes includes nonsynonymous mutations in *aceF*, *algU*, *bifA*, *gyrA*, *gyrB*, *lasR*, *mexA*, *mexB*, *mexZ*, *morA*, *mucA*, *nfxB*, *pvdS*, and *retS*.

Furthermore, several other of the mutations accumulated in lineages IT01, IT02, IT03, IT05, and IT06 may as well confer a selective advantage. For example, the exact C to T *phuR* promoter mutation (PAO1 genome position 5289158) found in isolates F23, F40, and F41 has previously been shown to increase the expression of the *Pseudomonas* heme uptake (phu) system; an adaptive trait shown to improve the uptake of iron from host hemoglobin [19]. Also, loss-of-function mutations in *rpoN* have frequently been found to be typical markers of CF lung infection [10], and in this study we found an *rpoN*(11-12insCT) frameshift mutation in IT02 strains H24 and H25.

Finally, we identified an A2058G mutation and a C2611T (*Escherichia coli* numbering) mutation in the 23S rRNA gene in the IT02 lineage (isolates H24 and H25) and the IT06 lineage (isolates F23, F40, and F41), respectively. The exact mutations have previously been shown to confer macrolide resistance in *P. aeruginosa* [20]. Thus, we hypothesize that the mutations have been selected due the use of azithromycin in clinic (see Methods section for description of antibiotic therapy), and in accordance with this the mutations were not observed until after the start of oral azithromycin treatment of the respective patients.

### Genetic relationship between IT01-IT06 lineages and other P. aeruginosa strains

Next, we sought to determine the genetic relationship between lineages IT01-IT06 and other genome sequenced strains of *P. aeruginosa*. We *de novo* assembled the genome of the earliest isolate(s) of each of the six clonal lineages and aligned the genomes to a panel of 60 genome sequences of other strains of *P. aeruginosa*. The panel consisted of eight completed genomes of *P. aeruginosa* reference strains (PAO1 [23], PA14 [24], LESB58 [25], PA7 [26], DK02 [21], 2192 and C3719 [27], PACS2), and 53 incomplete draft genomes of strains DK01-DK53, which we recently genome sequenced in a study of Danish CF patients [9].

**Fig. 3:**
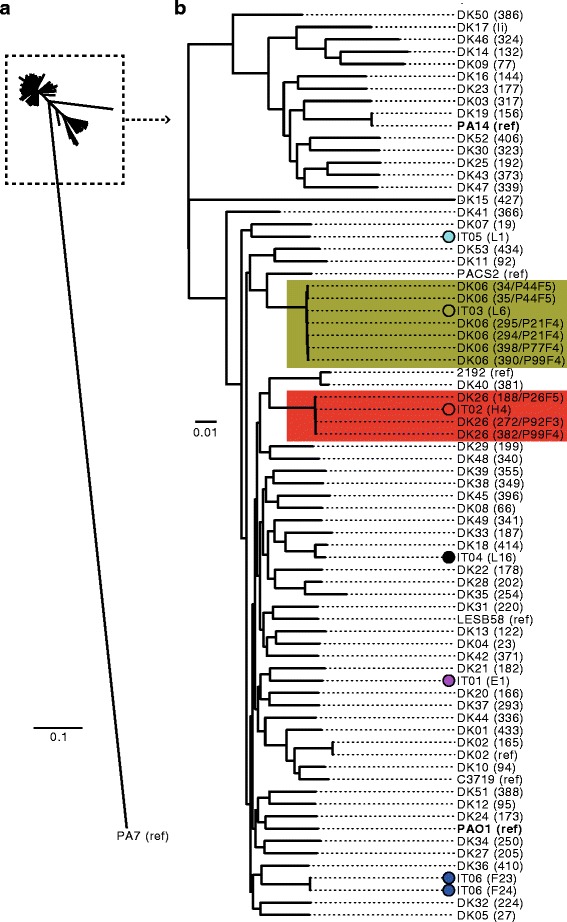
Phylogenetic tree based on core-genome alignment of different *Pseudomonas aeruginosa* strains. Genomes of the earliest isolate(s) of each of the lineages IT01-IT06 were aligned against a panel of 60 genome sequences of other strains of *P. aeruginosa*. **a** Tree showing the phylogeny of all isolates. **b** Subset of tree showing phylogeny of all isolates, except the outlier isolate PA7. Strains are either named after their lineage (IT01-IT06 or DK01-DK53) followed by the name of the specific isolate in parenthesis, or by the name of the reference strains (strains with completed genome sequences) followed by ‘ref’ in parenthesis. Phylogenetic tree was constructed using Harvest [28]

Using the Harvest suite [28], we aligned the panel of genomes and constructed a phylogenetic tree based on 188,727 SNPs identified in the core genome of the aligned strains (Fig. 3). Clustering of genomes based on core genome SNPs confirmed previous findings that, except for a few outlier strains (e.g. PA7), strains group into two major phylogenetic clusters containing strains PAO1 and PA14, respectively (Fig. 3a) [29, 30]. We observed no evidence that the geographical linked strains IT01-IT06 grouped more closely in the phylogenetic tree. However, we noted that strains IT01-IT06 all belong to the phylogenetic cluster containing strain PAO1 (Fig. 3a).

### Genetic distances between closely related isolates from different countries

We found that the IT02 and IT03 lineages were closely related to isolates of the DK26 and DK06 clone types, respectively (Fig. 3b), which have been isolated from Danish CF patients [9]. The close genetic relationship was also supported by multilocus sequence typing (MLST) analysis, showing the IT02/DK26 strains to be of sequence type ST-27 and the IT03/DK06 strains to be of sequence type ST-17.

**Fig. 4:**
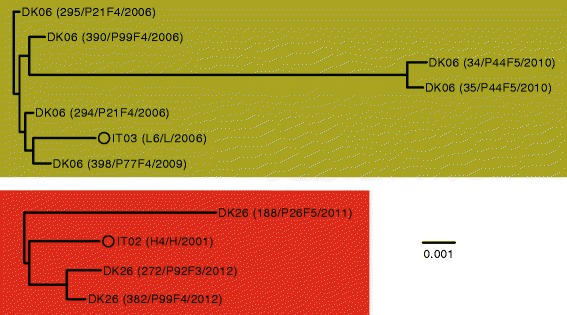
Phylogenetic trees based on core-genome alignment of *Pseudomonas aeruginosa* strains of the IT02/DK26 and IT03/DK06 clonal complexes. Phylogenetic trees were constructed using Harvest [28]. Name, patient origin, and year of isolation are shown for each isolate in parenthesis

To further investigate these two cases, we repeated the phylogenetic analysis, now only including the relevant isolates (Fig. 4). Note, since both clone types DK06 and DK26 were found in multiple patients in the study of the Danish patients, we included the earliest isolate from all the respective patients in our analysis (DK06 and DK26 isolates were from years 2006–2010 and 2011–2012, respectively). Interestingly, we found that more diversity was present between isolates from Denmark relative to the diversity present between isolates from Italy and Denmark (Fig. 4). For example, only 47 SNPs separated the Italian isolate H4 from isolate 294 sampled from a Danish CF patient (P21F4), whereas Danish isolates of the same clone type were different by up to 265 SNPs (isolates 294 and 34) (Table 2).

**Table 2:** Genetic distances (SNPs) between *Pseudomonas aeruginosa* strains

IT02/DK26	188/P26F5	272/P92F3	382/P99F4	H4			
188/P26F5	0	197	108	204			
272/P92F3	197	0	41	108			
382/P99F4	193	41	0	96			
H4	204	108	96	0			
IT03/DK06	294/P21F4	295/P21F4	34/P44F5	35/P44F5	398/P77F4	390/P99F4	L6
294/P21F4	0	18	265	259	21	26	47
295/P21F4	18	0	256	258	28	25	58
34/P44F5	265	256	0	23	259	262	277
35/P44F5	259	258	23	0	263	260	277
398/P77F4	21	28	259	263	0	26	53
390/P99F4	26	25	262	260	26	0	64
L6	47	58	277	277	53	64	0

### Host adaptation is associated with reduction in metabolic capacity

**Fig. 5:**
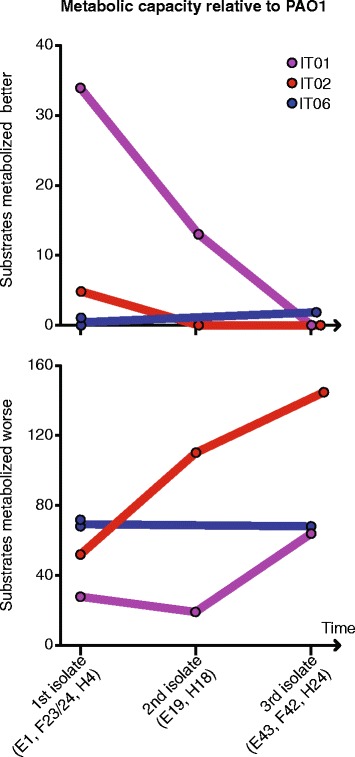
Changes in metabolic capacities over time relative to *Pseudomonas aeruginosa* reference strain PAO1. Number of substrates metabolized better or worse than PAO1 was measured using Biolog Phenotype MicroArrays (Biolog, Hayward, CA)

Finally, we sought to investigate the phenotypic changes that occurred during the course of infection. Reductions in metabolic capacity, for example caused by *rpoN* mutations, have previously been associated with host adaptation [5, 22], so we used phenotype microarrays (Biolog) to characterize the metabolic capacity of three strains from each of the lineages IT01, IT02, and IT06. The metabolic capacities of isolates of both lineages IT01 and IT02 decreased over time (19 and 10 years, respectively), whereas no change was observed for lineage IT06 over 4 years of infection (Fig. 5).

Mutants with loss-of-function mutations in the CbrAB two-component system is known to be unable or poor at utilizing a large range of carbon and nitrogen sources [31]. Therefore, we speculate that two nonsynonymous mutations accumulated in *cbrAB* may explain the loss of metabolic capacity in isolates H18 and H24 of the IT02 lineage.

In the IT01 lineage, several mutations may explain the reduced metabolic capacities of isolates E19 and E43 from years 2006 and 2012, respectively, relative to the ancestral isolate E1 from 1993. Both isolates E19 and E43 accumulated nonsynonymous mutations in genes *aceF*, *dgcB*, *gcdH*, *glcD*, *glnD*, and *vanA* belonging to PseudoCap function classes ‘Amino acid biosynthesis and metabolism’ and/or ‘Carbon compound catabolism’. In addition, isolate E43 harbor mutations in genes *cysB*, *pdxJ*, PA4910, *sdhA* that are involved in biosynthesis of amino acids, biosynthesis of cofactors, amino acid transport, and central metabolism, respectively. We suggest that the mentioned mutations may explain the reduction of metabolic capacity in the IT01 lineage.

### Changes in mucoidity, motility, ciprofloxacin susceptibility, and protease production

Besides reduction in metabolic capacity, the CF lung infection is associated with the appearance of a number of other phenotypes of which many are not usually observed among environmental, wild type isolates of this species. For example, mucoid colony formation, reduction in secretion of extracellular proteases, loss of flagella dependent motility, and antibiotic resistance are characteristics of isolates from chronic CF infections [32–34].

We phenotypically characterized the 25 isolates of clone types IT01-IT03 and IT05-06, and in accordance with previous studies, we found that nearly all isolates exhibited reduced swimming and swarming motility and protease secretion relative to the wild type-like reference strain PAO1 [35] (Fig. 2). The only isolates showing swimming and/or swarming capabilities better than PAO1 were L11, F4, and H1. Furthermore, three of the patients (E, F, and H) harbored mucoid isolates, and the two latest isolates of the IT02 lineage (H24 and H25) were resistant to ciprofloxacin.

The relative large numbers of mutations that appear in each of the clonal lineages make it difficult to associate specific mutations with phenotypic changes. Nonetheless, only mutations GacS(L309Q) and GacA(Y186stop), respectively, discriminated isolates F4 and H1 from the other isolates of the IT05 lineage. In agreement with literature [36, 37], this suggest that the mutations in the GacAS two-component regulatory system is the cause of increased motility and decreased extracellular protease activity of isolates F4 and H1. Furthermore, we suggest that a GyrA(T83I) mutation, which has previously been associated with ciprofloxacin resistance [38], explains ciprofloxacin resistance observed in isolates H24 and H25. Also, we suggest that mucoidity of isolates E37 and E42 may be caused by a nonsense mutation in *mucA* present in these two, but no other, isolates of the IT01 lineage.

In the IT02 lineage, a reduction of extracellular protease activity in isolates H18, H24, and H25 coincides with the fixation of a missense mutation in *lasR* encoding a regulator required for the expression of virulence-associated extracellular proteases LasA and LasB [39, 40]. In the same isolates, we suggest a loss of swimming motility to be caused by a missense mutation in *fleQ* encoding a positive regulator of flagella biosynthesis genes [41].

## Discussion

We have gained insight into *P. aeruginosa* infections using a collection of isolates sampled over 19 years from four Italian CF patients. We sequenced the genomes of 26 of the isolates and correlated identified mutations to changes in relevant phenotypes and antibiotic treatment used in the clinic. While there are several other clinical collections of *P. aeruginosa* that have been genome sequenced [2], this is the first genome analysis of isolates from Italian CF patients. We therefore anticipate that our study may serve as a reference for future research and helps to obtain a more complete basis for understanding *P. aeruginosa* infections in CF patients.

We found the genomic evolution of lineages from Italian patients to resemble the evolution of *P. aeruginosa* lineages from other countries. This included similar observed rates of mutation and evidence for host-associated selection for mutations in pathoadaptive genes. Accordingly, our results support previous findings of significant mutational signature of positive selection in relatively few pathoadaptive genes in contrast to neutral change in the large remainder of genes. Inter-study parallelism of mutations in the same pathoadaptive genes may by part be driven by similarities in antibiotic treatment regimes. However, we also found parallelism of mutations in genes that are not directly associated to antibiotic resistance (e.g. *aceF*, *bifA*, *morA*, and *retS*), and this may reflect similarities in host-dependent selective forces.

Unlike other studies [5, 8, 9, 42–44], we did not identify hypermutable lineages. Since our study only encompasses four patients, the lack of hypermutators may be accidental, and hypermutators may eventually appear as these are observed more frequently in late stage infections [42].

Patients were predominantly infected with strains unique to the particular patient. One exception was clone type IT05 which was shared by three patients. Despite of IT05’s occurrence in multiple patients, the IT05 clone type resembled a wild type phenotype, and this may explain why other clone types with typical host-associated phenotypes replace IT05 in all patients. As such, the pattern of strain replacement may be due to initial infections from a strain that reside in a common environmental source to which the patients are frequently exposed, and that this strain is subsequently replaced by other more rare, but also more fit, strains.

While other studies have given insight into *P. aeruginosa* within-host population diversity [5, 7, 45, 46], further investigations are needed to make conclusions about the diversity of *P. aeruginosa* in our four patients. However, we conducted an inter-clonal genomic analysis to determine the genetic relationship between lineages IT01-IT06 and a panel of 60 genome sequenced strains of *P. aeruginosa* from other countries. Hereby, we showed that lineages IT02 and IT03 were closely related to lineages DK26 and DK06, respectively, which have been isolated from Danish CF patients [9]. Since more diversity was present between isolates from Denmark relative to the diversity present between isolates from Italy and Denmark, our findings show that one must be cautious of using genome analysis to infer the country origin of lineages.

## Conclusions

This is the first whole-genome analysis of *P. aeruginosa* isolated from Italian CF patients, and together with both phenotypic and clinical information this dataset facilitates a better understanding of *P. aeruginosa* within-host genomic evolution, transmission, and population genomics. This may help the design of future intervention strategies for the clinical setting.

## Methods

### Bacterial isolates

The 35 *P. aeruginosa* isolates used in this study originates from the bacterial collection available at the Tuscan Regional Referral Center for Cystic Fibrosis in Florence, Italy. At this center, *P. aeruginosa* isolates sampled from CF patients have been stored twice a year since 1993. All patients enrolled in the study were in follow-up according to published guidelines [47, 48]. CF diagnosis was based on clinical features of the disease and concentration of chloride in sweat >60 mmol/liter [49]. Patients were regularly examined every 3 months. Data regarding their weight, height, body mass index (BMI), forced expiratory volume in one second (FEV_1_), microbiological status (including antibodies against *P. aeruginosa*) and antibiotic treatments were stored in the database. Cough swabs or sputum samples were processed following the national and international guidelines [50] (https://www.cysticfibrosis.org.uk/media/82034/CD_Laboratory_Standards_Sep_10.pdf).

The patients gave written consent to participate and for publication of their details. The study and use of bacterial isolates has been approved by the local ethics committee at the Department of Paediatric Medicine, Anna Meyer Children's University Hospital, Florence, Italy (approval no. 210).

### Antibiotic therapy principles in the clinic

Early eradication treatment was started at first detection of *P. aeruginosa* by administration of oral ciprofloxacin in combination with either nebulized colistin for three weeks or three months or inhaled tobramycin [48, 51, 52]. When *P. aeruginosa* reappeared after eradication therapy, treatment with oral ciprofloxacin and inhaled antibiotics was restarted.

At development of chronic *P. aeruginosa* infection [53], intravenous antibiotic treatment was administered together with inhaled colistin or inhaled tobramycin. Patients also received treatments with oral azitromycin [54]. During follow-up, in the case of mild pulmonary exacerbation, all patients were treated with oral ciprofloxacin in combination with inhaled antibiotics. Severe pulmonary exacerbations were treated with parenteral antibiotic treatments (ceftazidime or meropenem in combination with tobramycin once a day) according to suggested dosage [47].

### Patient information

Patient E (cystic fibrosis transmembrane conductance regulator gene (*CFTR*) genotype F508del/F508del) had his first *P. aeruginosa* colonization at the age of 19 months and developed chronic infection at the age of three years. He remained stable without pulmonary exacerbation until 2012. Due to excellent lung function (FEV_1_ = 115 % of predicted) he was treated only intermittently with oral ciprofloxacin in combination with inhaled antibiotics. Oral azithromycin was started in 2006.

Patient F (*CFTR* genotype 711 + 19 A/T /3272-9 A/t) had her first *P. aeruginosa* colonization at the age of 33 months and developed chronic infection at the age of four years. She had a stable FEV_1_ (60 %) until 2008, when she began a progressive clinical deterioration with FEV_1_ (20 %) in 2012. She was frequently treated with high doses of ciprofloxacin with inhaled antibiotics from 2001 until 2010, with repeated parenteral antibiotic cycles with ceftazidime or meropenem in association with tobramycin once a day. Oral azithromycin was started in 2004.

Patient H (*CFTR* genotype F508del/F508del) had his first *P. aeruginosa* colonization at the age of 8 months and was chronically infected at the age of 1 year. After chronic *P. aeruginosa* infection was developed, he was treated with fluoroquinolones and tobramycin by inhalation. Oral quinolones were used to treat mild pulmonary exacerbations. He underwent parenteral antibiotic treatment (ceftazidime and tobramycin) twice from 2011 to 2013 for severe pulmonary exacerbations. His lung function declined from 2006 (FEV_1_ = 80 %) to 2011 (FEV_1_ ≤ 40 %). Oral azithromycin was started in 2005. In addition, tetracyclines, trimethoprim-sulfamethoxazole, and linezolid were used to treat methicillin-resistant *Staphylococcus aureus*.

Patient L (*CFTR* genotype F508del/N1303K) had her first *P. aeruginosa* colonization at the age of 27 months. She developed chronic infection at the age of 20 years. However, her clinical conditions were stable and she had good lung function (FEV_1_ = 80 %). After chronic *P. aeruginosa* lung infection developed, she was treated with antibiotics by inhalation. She used oral quinolones for mild pulmonary exacerbations together with parenteral antibiotic treatment (ceftazidime and tobramycin), which was necessary twice in the period 2011 to 2013. She did not tolerate treatment with oral macrolides.

### ArrayTube genotyping

*P. aeruginosa* genotypes were determined using an ArrayTube multimarker microarray targeting 13 SNPs in the core genome and additional genetic markers in the accessory genome (Clondiag Chip Technologies, Germany) [13]. The ArrayTube genotyping assay was performed according to the protocol provided by the manufacturer.

### Genome sequencing

Genomic DNA was prepared from *P. aeruginosa* isolates on a QIAcube system using a DNeasy Blood and Tissue Kit (Qiagen). Genomes were sequenced by BGI (Shenzhen, China) to an average coverage depth of at least 75-fold (range 75- to 139-fold) on an Illumina HiSeq2000 platform generating 100-nt paired-end reads.

### Mutation detection and construction of phylogenetic trees

Mutations that had accumulated in *P. aeruginosa* lineages IT01-IT03 and IT05-IT06, were identified as previously described (without any modifications) [9]. Based on the identified SNPs, we computed maximum-parsimonious phylogenetic trees using PAUP* version 4.0b10 [55]. Consistency indexes were calculated as the number of mutations divided by the minimum number of mutational events required to explain phylogenies. The consistency index will equal one when there is no homoplasy.

### Estimation of mutation rates

Bayesian analysis of evolutionary rate was performed using BEAST, version 1.7.0 [56], with a lognormal relaxed molecular clock model and a general time-reversible substitution model. Mutation rate of the IT01 lineage was calculated from a chain length of 50 million steps, sampled every 5,000 steps. The first 5 million steps were discarded as a burn-in. The ESS of all parameters were >1,000 as calculated by Tracer, version 1.5 (available from http://beast.bio.ed.ac.uk/Tracer), which was also used to calculate the 95 % HPD confidence intervals of the mutation rate (i.e. an interval within which the modeled parameter resides with 95 % probability). Mutation rates of the other lineages (IT02, IT03, IT05, IT06) could not be estimated since the number and temporal distribution of isolates were insufficient to obtain proper estimates of the mutation rates (ESS of modeled parameters < 10).

### De novo assembly and inter-clonal whole-genome alignments

We *de novo* assembled the genome of the earliest isolate(s) of each of the six clonal lineages (E1, F23, F24, H4, L1, L6, L16) and genomes of representatives of strains DK01-DK53, which we recently genome sequenced in a study of Danish CF patients [9].

Sequence reads from each isolate were error corrected using ALLPATHS-LG’s stand-alone error correction tool [57] and *de novo* assembled using the de Bruijn graph-based assembler Velvet (version 1.2.08) [58]. For each sample several assemblies were run. This implies that for each dataset ‘velveth’ command was executed using *k*-mer sizes in the range of 35 to 95. Next, the ‘velvetg’ command was run using the parameters: min_contig_lgth = 400, exp_cov = auto, and scaffolding = no. Based on the number of contigs, the best cumulative rank for N50, and the length of the largest contig, the best *k*-mer size was selected and the exp_cov was noted. A final assembly was performed using the best *k*-mer size for velveth and with velvetg using the parameters: min_contig_lgth = 400, scaffolding = no, and the exp_cov that was calculated in the first ensemble of assemblies. The size range of the *de novo* assembled genomes of strains of lineages IT01-06 was 6.3-6.9 Mbp, and each genome shared 95.8–97.0 % of the content *P. aeruginosa* reference strain PAO1.

*De novo* assembled genomes and completed genomes from the public domain [59] were aligned against each other using the Harvest suite [28] with default parameters using all genomes (option ‘–c’ set to ‘YES’) and with genome sequence of strain PAO1 as reference.

MLST sequence types were determined using a public available online tool developed for use on *de novo* assembled genomes [60]. Sequence types of lineages IT01-06 were ST-111, ST-27, ST-17, ST-389, ST-1748, and ST-348, respectively.

### Biolog phenotype profiling

Phenotype MicroArray (Biolog, Hayward, CA) experiments were performed in duplicate according to the manufacturer’s instructions [61, 62]. *P. aeruginosa* strains were streaked on LB agar plates and incubated at 37 °C until colonies appeared on the plates (16–30 hours (h)). Cells were swabbed from the plates and suspended in IF-0 GN Base (inoculation fluid) at a density corresponding to 42 % transmittance in the Biolog turbidimeter. The cell suspensions were diluted 1:6 in IF-0 minimal medium containing Biolog redox dye mixture D (tetrazolium), and 100-μL aliquots were added to carbon-source plates (PM1 and PM2A). For the nitrogen-source plate (PM3B), inoculations were supplemented with 30 mM glucose and 2 μM ferric citrate. The plates were incubated at 37 °C in an OmniLog plate reader (Biolog) for 72 h, and growth/respiration was measured kinetically by determining the colorimetric reduction of tetrazolium dye. Export of OmniLog data was performed using OmniLog OL_FM/Kin 1.20.02 software (Biolog). The average area beneath each kinetic curve was used for analysis. OD600 was measured after 60 h of growth at 37 °C, and relative catabolic capacities of individual substrates were determined as previously described [63].

### Motility assays

The motility of all isolates was tested on ABT minimal medium supplemented with 0.5 % Casamino acids and 0.5 % glucose as previously described [64]. Briefly, agar plates were inoculated with single colonies on the top or in the middle of the plates to measure swarming or swimming motility, respectively. Swimming and swarming were measured on 0.3 % (wt/vol) and 0.6 % (wt/vol) agar, respectively, with incubation for 24 h at 30 °C. The motility zones in triplicates were measured relative to the motility of the reference strain PAO1.

### Skim milk protease assay

The production of protease was determined by applying one colony of the isolate grown on Luria-Bertani (LB) medium agar plates to skim milk agar plates (LB agar with 10 % skim milk). The plates were incubated for 24 h at 37 °C, and the clearing zones were measured.

### Ciprofloxacin susceptibility testing

Ciprofloxacin susceptibility was tested by the Bauer-Kirby agar disk diffusion method. Discs (Neo-Sensitabs) were purchased from Rosco Diagnostica (Taastrup, Denmark). The diameters of clearing zones were measured in millimeters (mm) after 20 h at 37 °C.

## Availability of data and materials

Sequence reads from all *P. aeruginosa* isolates are deposited in the Short Read Archive under accession number PRJEB8020 [EMBL:PRJEB8020].

## Abbreviations

BMI: Body mass index
CF: Cystic fibrosis
CFTR: Cystic fibrosis transmembrane conductance regulator gene
dN: Rate of nonsynonymous genetic changes
dS: Rate of synonymous genetic changes
ESS: Effective sample size
FEV_1_: Forced expiratory volume in one second
HPD: Highest posterior density
h: Hour
mm: Milimeter
MLST: Multilocus sequence typing
Phu: *Pseudomonas* heme utilization
SNP: Single nucleotide polymorphism
